# The abnormal accumulation of pathological proteins and compensatory functional connectivity enhancement of insula subdivisions in mild cognitive impairment

**DOI:** 10.3389/fnagi.2025.1506478

**Published:** 2025-03-18

**Authors:** Darui Zheng, Chen Xue, Yingcai Feng, Yiming Ruan, Wenzhang Qi, Qianqian Yuan, Zonghong Li, Chaoyong Xiao

**Affiliations:** ^1^Department of Radiology, The Affiliated Brain Hospital of Nanjing Medical University, Nanjing, China; ^2^The First Clinical Medical College of Nanjing University of Chinese Medicine, Nanjing, China

**Keywords:** mild cognitive impairment, Amyloid-beta, tau protein, insular subdivisions, functional connectivity, functional magnetic resonance imaging

## Abstract

**Background:**

The insula is a critical node of the salience network responsible for initiating network switching, and its dysfunctional connections are linked to the mechanisms of mild cognitive impairment (MCI). This study aimed to explore the changes in functional connectivity (FC) of insular subregions in MCI patients with varying levels of cerebrospinal fluid (CSF) pathological proteins, and to investigate the impact of these proteins on the brain network alterations in MCI.

**Methods:**

Based on CSF Amyloid-beta (Aβ, A) and phosphorylated tau protein (p-tau, T), MCI patients were classified into 54 A−T−, 28 A+T−, and 52 A+T+ groups. Seed-based FC analysis was employed to compare the FC differences of insular subregions across the three groups. Correlation analysis was further conducted to explore the relationship between altered FC and cognitive function. Finally, ROC curve analysis was used to assess the diagnostic value of altered FC of insular subregion in distinguishing between the groups.

**Results:**

In the left ventral anterior insula, left dorsal anterior insula, and bilateral posterior insular subnetworks, both the A+T− and A+T+ groups showed increased FC compared to the A−T− group, with the A+T+ group showing further increased FC compared to the A+T− group. Additionally, FC of the left cerebellar posterior lobe was negatively correlated with RAVLT-learning, and FC of the left middle frontal gyrus was negatively correlated with p-tau levels. Finally, logistic regression analysis demonstrated that multivariable analysis had high sensitivity and specificity in distinguishing between the groups.

**Conclusion:**

This study showed that MCI patients with abnormal CSF pathological protein levels exhibit compensatory increases in FC of insular subregions, which in turn affect cognitive function. Our findings contributed to a better understanding of the pathophysiology and underlying neural mechanisms of MCI.

## Introduction

The core symptom of mild cognitive impairment (MCI) is cognitive decline, which often represents the prodromal stage of Alzheimer’s disease (AD) (Jessen et al., 2014; Xue et al., 2019). The progression rate from MCI to AD varies among studies, with an average annual rate of 10%–15% (Petersen et al., 2001; Guo et al., 2012). Over a span of 6 years, more than 80% of individuals with MCI have been observed to eventually develop AD (Busse et al., 2006). Understanding the pathological mechanisms of MCI, along with early diagnosis and timely intervention, is crucial for potentially reversing its progression (Kim et al., 2021). Studies have shown that various biomarkers can predict the development of MCI and AD, as well as the likelihood of MCI converting to AD years before clinical symptoms appear (Lin et al., 2020; Crystal et al., 2023). Consistent evidence indicates that the hallmark pathologies, Amyloid-beta (Aβ) and tau protein, are central to AD’s core pathology. Multiple studies have focused on the development of Aβ and tau biomarkers to track the progression of AD, revealing that Aβ deposition is one of the earliest events, followed by tau accumulation (Jack et al., 2013; Selkoe and Hardy, 2016; Jack et al., 2018). However, recent evidence suggests tau pathology can emerge independently of Aβ and may even precede Aβ deposition in certain brain regions (Silvestro et al., 2022). These findings highlight the complex interplay between Aβ and tau pathology in the progression of AD, suggesting that their mechanisms may involve multiple interwoven pathological pathways, thereby underscoring the need for further research. Therefore, understanding the relationship between Aβ, tau, and brain function is key to grasping the early functional changes in AD, providing valuable strategies for the early diagnosis and treatment of MCI.

Resting-state functional magnetic resonance imaging (fMRI) has been proven to be an effective method for analyzing complex neural networks by measuring intrinsic brain fluctuations in blood oxygen level-dependent signals (Biswal et al., 1995; Zhang and Raichle, 2010; Xue et al., 2020). The insula is a core region of the salience network (SN), involved in higher-order cognition, autonomic functions, emotional, and sensory processes (Naqvi and Bechara, 2010). The insula consists of several heterogeneous subregions, including the ventral anterior insula (vAI), dorsal anterior insula (dAI), and posterior insula (PI) (Yang et al., 2022; Zhao et al., 2023). The posterior insula is responsible for collecting and integrating various interoceptive signals, which are then relayed to the anterior insula for higher-order representation and perception processing. The anterior insula is associated with arousal, interoceptive awareness, and cognitive-emotional processing, with the dorsal anterior insula being more involved in higher cognitive functions. These distinct functional roles suggest that different insular subregions may contribute uniquely to cognitive, emotional, and sensory processing, making them critical targets for investigating network alterations in MCI and AD.

Previous studies have shown that different insular subregions exhibit distinct resting-state functional connectivity (FC) patterns and are part of different functional networks, which are differentially affected across AD spectrum (Deen et al., 2011; Zhang et al., 2016; Yang et al., 2022). Additionally, changes in the FC of insular subregions have been suggested to contribute to the cognitive, emotional, and sensory symptoms observed in the AD spectrum (Liu et al., 2018; Li et al., 2024; Tian et al., 2024). However, there is still a lack of systematic research on the role of Aβ and tau pathology in the functional organization of insular subregions in MCI patients. In particular, the specific impact of early AD pathological burden on the FC patterns of different insular subregions remains unclear. Therefore, investigating how Aβ and tau burden levels influence changes in insular FC in MCI patients will not only enhance our understanding of the role of pathological burden in neural communication and information transmission during the MCI stage but also provide potential neuroimaging biomarkers for the early identification of AD.

Therefore, the purpose of this study was to investigate the FC abnormalities of insular subregions in MCI patients with different cerebrospinal fluid (CSF) pathological protein levels and their correlation with cognitive function. We hypothesized that the presence of pathological proteins affects the FC of insular subregions and was associated with changes in cognitive function. These alterations were crucial for understanding the pathological mechanisms of MCI and predicting the levels of pathological proteins in MCI.

## Materials and methods

### Subjects

The data utilized in this study were all sourced from the Alzheimer’s Disease Neuroimaging Initiative (ADNI) database^1^. The ADNI database provided detailed inclusion and exclusion criteria for MCI. The present study included all baseline MCI patients with resting-state fMRI data from the ADNI-2 and ADNI-3 phases, totaling 241 participants. Due to partial data loss, 225 participants remained. After further excluding 13 participants with excessive head motion (cumulative translation or rotation of > 3.0 mm or 3.0°), a final total of 212 participants were included in the analysis. The specific MCI diagnostic and inclusion criteria can be found in the *SI Methods*. Following previous studies, a threshold of < 977 pg/ml was applied to identify abnormal CSF Aβ_42_, and > 24 pg/ml was used to identify abnormal CSF phosphorylated tau protein (p-tau) (Hansson et al., 2018; Vromen et al., 2023). Based on these criteria, the MCI patients were categorized including abnormal Aβ_42_ and p-tau (A+T+), abnormal Aβ_42_ and normal p-tau (A+T−), normal Aβ_42_ and abnormal p-tau (A−T+), and normal Aβ_42_ and p-tau (A−T−). The A−T+ group was excluded due to their classification outside the AD spectrum according to the A/T/N framework (Yoon et al., 2022). As a result, the present study included 54 A−T− participants, 28 A+T− participants, and 52 A+T+ participants.

Ethical approval for the ADNI study was granted by the institutional review committees of all participating institutions. Written informed consent was obtained from participants or their authorized representatives. Further information was available on the ADNI website^2^.

### Cognitive function

To evaluate cognitive function, we conducted comparisons between groups using the composite scores for episodic memory (EM) and executive function (EF). Additional information on EM and EF were provided in *SI Methods.*

### Pathological sample acquisition

The CSF samples were collected per the Alzheimer’s Association Flow Chart for CSF biomarkers. The INNO-BIAALZBio3 immunoassay kit was used to determine CSF levels of Aβ_42_, total tau protein (t-tau), and p-tau. In subsequent statistical processing, if the value of Aβ_42_ exceeds 1,700, it will be treated as 1,700 for statistical purposes.

### MRI data acquisition

Detailed scanning information can be obtained from http://adni.loni.usc.edu/wp-content/uploads/2010/05/ADNI2 MRI Training-Manual-FINAL.pdf and http://adni.loni.usc.edu/wp-content/uploads/2017/07/ADNI3-MRI-protocols.pdf.

### Functional data preprocessing

Preprocessing of resting-state fMRI data was performed using Data Processing and Analysis for Brain Imaging (DPABI^3^) software and its Data Processing Assistant for Resting-State fMRI (DPARSF) module in MATLAB 2021b^4^ (Yan et al., 2016). Details regarding image preprocessing were provided in ***SI Methods***.

### FC analysis

Seed-based FC analysis was carried out to explore the alternation of insula subdivisions using the DPABI software. According to previous study, six 6 mm spherical region of interest (ROI) were created, including left vAI (MNI space: −33, 13, −7), right vAI (MNI space: 32, 10, −6), left dAI (MNI space: −38, 6, 2), right dAI (MNI space: 35, 7, 3), left PI (MNI space: −38, −6, 5) and right PI (MNI space: 35, −11, 6) (Lu et al., 2020). The mean time series of each ROI was extracted as the reference time course, and voxel-wise Pearson correlation analysis was performed between each ROI and the entire brain within the gray matter mask. Then, a Fisher’s r-to-z transformation was performed to improve the normality. Finally, for each subject, we generated six z-score maps that represented the intrinsic FC patterns of the six insular subregions.

### Statistical analysis

Statistical analyses were conducted using Statistical Package for the Social Sciences (SPSS) software, version 25.0 (IBM, Armonk, New York, NY, United States). ANOVA and chi-square tests were applied to compare demographic characteristics, neurocognitive scales, and CSF pathological protein levels across the three groups: A−T−, A+T−, A+T+. *Post hoc* comparisons were adjusted using Bonferroni correction, with statistical significance set at *p* < 0.05.

A one-way ANOVA analysis was performed to assess differences in FC of each insular subregion after controlling for the influence of age, gender, and years of education (GRF corrected, voxel *p* < 0.005, cluster *p* < 0.05). Subsequently, *post hoc* comparisons were conducted using two-sample *t*-tests, with the resulting mask from the ANOVA analysis with age, gender, and years of education as covariates (GRF corrected, voxel *p* < 0.005, cluster *p* < 0.05).

Correlation analyses were carried out in SPSS to explore relationships between altered FC of each insular subregion and cognitive function, as well as CSF pathological proteins, while adjusting for age, sex, and years of education as covariates (*p* < 0.05).

### Binary logistic regression analysis

Univariate and multivariable binary logistic regression were performed using SPSS software to evaluate the diagnosis value of altered FC of each insular subregion in A+T− and A+T+ groups. Altered FCs identified in the univariate analysis were included in the multivariable models through backward elimination, based on the likelihood ratio with a variable selection criterion of *p* < 0.05. The predictive performance of the univariate and multivariable models was assessed using the receiver operating characteristic (ROC) curve and the area under the ROC curve (AUC), with results presented in terms of accuracy, sensitivity, and specificity.

## Results

### Demographic and neurocognitive characteristics

As shown in Table 1, the A+T+ group was older than both A−T− and A+T− groups. As expected, compared to the A−T− group, the A+T+ group showed lower scores in the immediate recall and learning components of Rey Auditory Verbal Learning Test (RAVLT), and the composite EM score (Bonferroni corrected, *p* < 0.05).

**TABLE 1:** Demographics and clinical measures of three groups, including A−T−, A+T−, and A+T+.

	A−T− (54)	A+T− (28)	A+T+ (52)	F values (χ^2^)	*P*=values
Age (years)	68.97 (7.65)	71.68 (7.30)	73.16 (5.79)**	4.982	0.008^a^
Gender (F/M)	20/34	13/15	20/32	0.723	0.697
Years of education	15.91 (2.64)	16.46 (2.57)	16.33 (2.65)	0.534	0.588
MMSE	28.06 (1.89)	28.18 (1.74)	27.38 (2.18)	2.100	0.127
MoCA	23.79 (3.06)	23.04 (2.52)	22.76 (3.61)	1.402	0.250
RAVLT-immediate	38.43 (9.42)	34.68 (9.21)	33.10 (8.94)*	4.620	0.012^a^
RAVLT-learning	4.91 (2.24)	4.36 (2.09)	3.73 (2.39)*	3.559	0.031^a^
RAVLT-forgetting	4.43 (3.93)	4.57 (2.17)	5.10 (2.51)	0.654	0.522
RAVLT-prec-forgetting	46.08 (56.25)	57.15 (29.42)	65.50 (30.51)	2.757	0.067
EM	0.49 (5.20)	0.30 (1.05)	0.05 (0.65)**	5.113	0.007^a^
EF	0.65 (0.91)	0.23 (1.05)	0.24 (0.84)	3.310	0.040
Aβ_42_	1484.25 (255.03)	726.19 (199.64)***	622.09 (159.11)***	249.840	< 0.001^ab^
T-tau	201.89 (44.35)	178.88 (46.61)	383.71 (130.72)***/***	73.112	< 0.001^ac^
P-tau	17.36 (3.98)	16.30 (4.68)	40.58 (16.54)***/***	75.415	< 0.001^ac^

*^a^Post hoc* analyses showed a significantly group difference between A+T+and A−T−.

*^b^Post hoc* analyses showed a significantly group difference between A+T− and A−T−.

*^c^Post hoc* analyses showed a significantly group difference between A+T+ and A+T−.

*p < 0.05;

**p < 0.01;

***p < 0.001; A+T+, abnormal Aβ_42_ and p-tau; A+T−, abnormal Aβ_42_ and normal p-tau; A−T−, normal Aβ_42_ and p-tau; Aβ, Amyloid-beta protein; p-tau, phosphorylated tau protein; t-tau, total tau protein. Numbers are given as means (standard deviation, SD) unless stated otherwise. MMSE, Mini-mental State Examination; MoCA, Montreal Cognitive Assessment; RAVLT, Rey Auditory Verbal Learning Test; EM, episodic memory; EF, executive function.

### FC analysis

**FIGURE 1:**
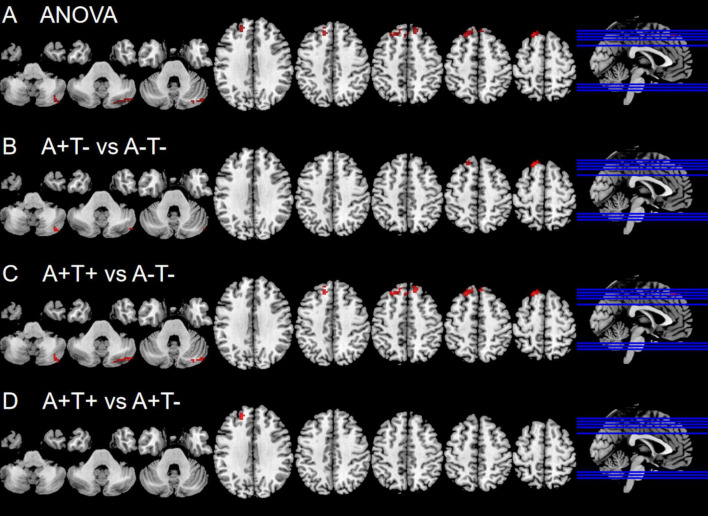
Brain regions exhibiting significant differences in functional connectivity of the left ventral anterior insula. **(A)** Significant differences in functional connectivity of the left ventral anterior insula among three groups, including A−T−, A+T−, and A+T+ (GRF corrected, voxel *p* < 0.005, cluster *p* < 0.05); **(B–D)** Results of *post hoc* analysis in voxel-wise analysis (GRF corrected, voxel *p* < 0.005, cluster *p* < 0.05). A+T+, abnormal Aβ_42_ and p-tau; A+T−, abnormal Aβ_42_ and normal p-tau; A−T−, normal Aβ_42_ and p-tau.

In the left vAI subnetwork, the ANOVA analysis showed significant alterations in FC across three groups, including the right cerebellum posterior lobe (CPL), and bilateral superior frontal gyrus (SFG). Compared to A-T- group, A+T− group showed increased FC in the right CPL and left SFG while A+T+ showed increased FC in the right CPL and left SFT. Compared to A+T− group, the A+T+ group showed increased FC in the left SFG (GRF corrected, voxel *p* < 0.005, cluster *p* < 0.05). These results were obtained while accounting for age, sex, and years of education (Figure 1 and Table 2).

**TABLE 2:** The differences of functional connectivity (FC) of left ventral anterior insula across three groups.

Region(aal)	Peak MNI coordinate			F/t	Cluster number
	**x**	**y**	**z**		
**ANOVA**					
R cerebellum posterior lobe	36	−72	−45	8.2293	55
B superior frontal gyrus	−18	33	57	11.5676	171
**A+T− vs A−T−**					
R cerebellum posterior lobe	42	−78	−42	3.5904	23
L superior frontal gyrus	−18	33	57	3.9076	20
**A+T− vs A−T−**					
R cerebellum posterior lobe	36	−72	−45	3.9446	55
L superior frontal gyrus	−18	33	57	4.4324	138
**A+T− vs A−T−**					
L superior frontal gyrus	−18	48	27	3.6113	31

The x, y, z coordinates is the primary peak locations in the MNI space. Cluster size > 10 voxels in ANOVA analysis, GRF corrected, voxel *p* < 0.005, cluster *p* < 0.05; Cluster size > 10 voxels in *post-hoc* test, GRF corrected, voxel *p* < 0.005, cluster *p* < 0.05; A+T+, abnormal Aβ_42_ and p-tau; A+T−, abnormal Aβ_42_ and normal p-tau; A−T−, normal Aβ_42_ and p-tau; B, bilateral; L, left; R, right.

**FIGURE 2:**
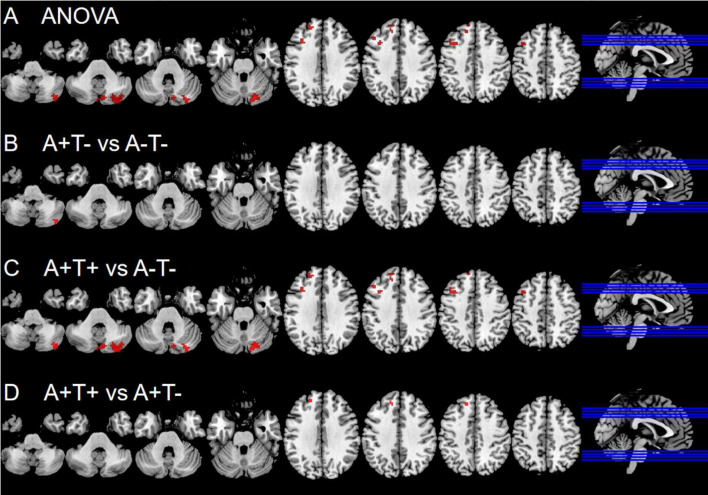
Brain regions exhibiting significant differences in functional connectivity of the left dorsal anterior insula. **(A)** Significant differences in functional connectivity of the left dorsal anterior insula among three groups, including A−T−, A+T−, and A+T+ (GRF corrected, voxel *p* < 0.005, cluster *p* < 0.05); **(B–D)** Results of *post hoc* analysis in voxel-wise analysis (GRF corrected, voxel *p* < 0.005, cluster *p* < 0.05). A+T+, abnormal Aβ_42_ and p-tau; A+T−, abnormal Aβ_42_ and normal p-tau; A−T−, normal Aβ_42_ and p-tau.

In the left dAI subnetwork, the ANOVA analysis showed significant alternations in FC across three groups, including the right CPL, left SFG and left middle frontal gyrus (MFG). Compared to A−T− group, the A+T− group showed increased FC in the right CPL while A+T+ group showed increased FC in the right CPL, left SFG, and left MFG. Compared to A+T− group, the A+T+ group showed increased FC in the SFG (GRF corrected, voxel *p* < 0.005, cluster *p* < 0.05). These results were obtained while accounting for age, sex, and years of education (Figure 2 and Table 3).

**TABLE 3:** The difference of functional connectivity (FC) of left dorsal anterior insula across three groups.

Region(aal)	Peak MNI coordinate			F/t	Cluster number
	**x**	**y**	**z**		
**ANOVA**					
R cerebellum posterior lobe	21	−78	−27	9.3634	177
L superior frontal gyrus	−18	45	30	7.791	44
L middle frontal gyrus	−42	15	45	9.6399	63
**2 vs 1**					
R cerebellum posterior lobe	39	−75	−42	3.1519	12
**3 vs 1**					
R cerebellum posterior lobe	21	−78	−27	4.2211	176
L superior frontal gyrus	−21	48	36	3.6149	35
L middle frontal gyrus	−39	15	45	3.965	60
**3 vs 2**					
L superior frontal gyrus	−15	39	42	2.9677	18

The x, y, z coordinates is the primary peak locations in the MNI space. Cluster size > 10 voxels in ANOVA analysis, GRF corrected, voxel *p* < 0.005, cluster *p* < 0.05; Cluster size > 10 voxels in *post-hoc* test, GRF corrected, voxel *p* < 0.005, cluster *p* < 0.05; A+T+, abnormal Aβ_42_ and p-tau; A+T−, abnormal Aβ_42_ and normal p-tau; A−T−, normal Aβ_42_ and p-tau; L, left; R, right.

**FIGURE 3:**
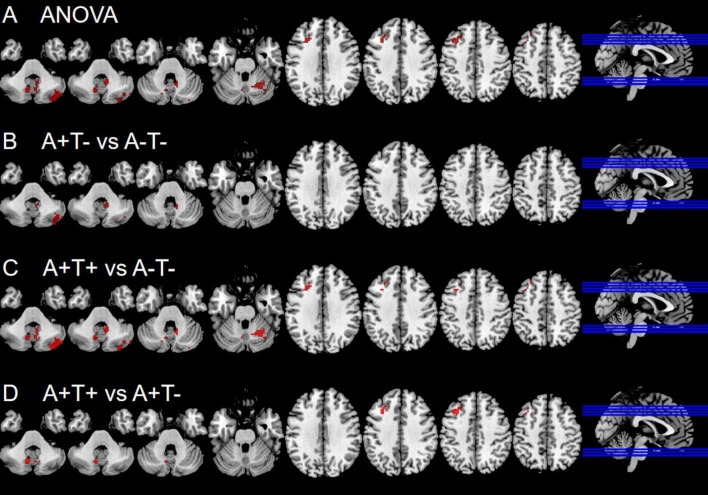
Brain regions exhibiting significant differences in functional connectivity of the left posterior insula. **(A)** Significant differences in functional connectivity of the left posterior insula among three groups, including A−T−, A+T−, and A+T+ (GRF corrected, voxel *p* < 0.005, cluster *p* < 0.05); **(B–D)** Results of *post hoc* analysis in voxel-wise analysis (GRF corrected, voxel *p* < 0.005, cluster *p* < 0.05). A+T+, abnormal Aβ_42_ and p-tau; A+T−, abnormal Aβ_42_ and normal p-tau; A−T−, normal Aβ_42_ and p-tau.

In the left PI subnetwork, the ANOVA analysis showed significant alternations in FC across three groups, including bilateral CPL and left MFG. Compared to A−T− group, the A+T− group showed increased right CPL and the A+T+ group showed increased FC in the bilateral CPL and left MFG. Compared to A+T− group, the A+T+ group showed increased FC in the bilateral CPL and left MFG (GRF corrected, voxel *p* < 0.005, cluster *p* < 0.05). These results were obtained while accounting for age, sex, and years of education (Figure 3 and Table 4).

**TABLE 4:** The difference of functional connectivity (FC) of left posterior insula across three groups.

Region(aal)	Peak MNI coordinate			F/t	Cluster number
	**x**	**y**	**z**		
**ANOVA**					
L cerebellum posterior lobe	−12	−60	−51	8.927	57
R cerebellum posterior lobe	9	−63	−48	10.6711	114
R cerebellum posterior lobe	42	−78	−42	11.6532	110
L middle frontal gyrus	−36	18	45	8.355	57
**2 vs 1**					
R cerebellum posterior lobe	42	−75	−42	5.3702	83
R cerebellum posterior lobe	9	−48	−36	3.2984	16
**3 vs 1**					
L cerebellum posterior lobe	−12	−60	−51	3.8807	56
R cerebellum posterior lobe	9	−63	−48	4.3974	114
R cerebellum posterior lobe	42	−78	−42	4.2264	110
L middle frontal gyrus	−27	33	48	3.4567	39
**3 vs 2**					
L cerebellum posterior lobe	−9	−66	−39	3.6638	39
R cerebellum posterior lobe	3	−66	−42	3.3305	11
L middle frontal gyrus	−33	24	42	3.7286	37

The x, y, z coordinates is the primary peak locations in the MNI space. Cluster size > 10 voxels in ANOVA analysis, GRF corrected, voxel *p* < 0.005, cluster *p* < 0.05; Cluster size > 10 voxels in *post-hoc* test, GRF corrected, voxel *p* < 0.005, cluster *p* < 0.05; A+T+, abnormal Aβ_42_ and p-tau; A+T−, abnormal Aβ_42_ and normal p-tau; A−T−, normal Aβ_42_ and p-tau; L, left; R, right.

**FIGURE 4:**
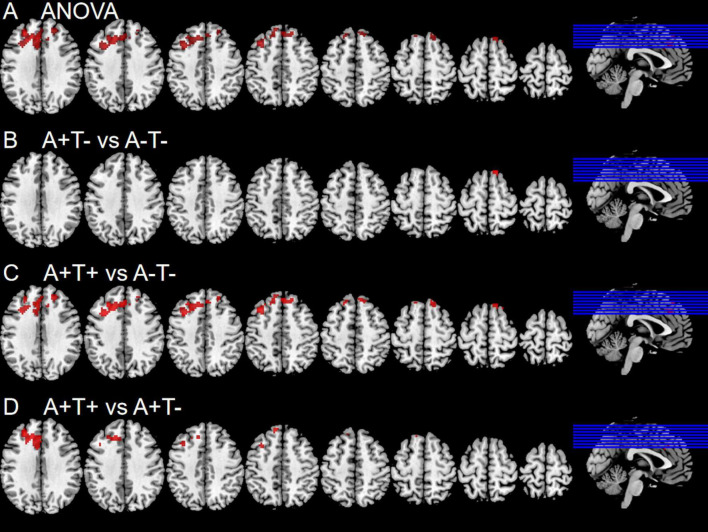
Brain regions exhibiting significant differences in functional connectivity of the right posterior insula. **(A)** Significant differences in functional connectivity of the right posterior insula among three groups, including A−T−, A+T−, and A+T+ (GRF corrected, voxel *p* <0.005, cluster *p* < 0.05); **(B–D)** Results of *post hoc* analysis in voxel-wise analysis (GRF corrected, voxel *p* < 0.005, cluster *p* < 0.05). A+T+, abnormal Aβ_42_ and p-tau; A+T−, abnormal Aβ_42_ and normal p-tau; A−T−, normal Aβ_42_ and p-tau.

In the right PI subnetwork, the ANOVA analysis showed significant alternations in FC across three groups, including bilateral MFG and SFG. Compared to the A−T− group, the A+T− group showed increased FC in the right SFG while the A+T+ group showed increased FC in the bilateral MFG. Compared to the A+T− group, the A+T+ group showed increased FC in the left MFG and left SFG (GRF corrected, voxel *p* < 0.005, cluster *p* < 0.05). These results were obtained while accounting for age, sex, and years of education (Figure 4 and Table 5).

**TABLE 5:** The difference of functional connectivity (FC) of right posterior insula across three groups.

Region(aal)	Peak MNI coordinate			F/t	Cluster number
	**x**	**y**	**z**		
**ANOVA**					
B middle frontal gyrus/superior frontal gyrus	−3	30	36	11.8022	571
**2 vs 1**					
R superior frontal gyrus	12	30	63	3.7761	21
**3 vs 1**					
B middle frontal gyrus	−36	21	45	4.2427	469
**3 vs 2**					
L middle frontal gyrus	−27	45	27	3.9375	210
L middle frontal gyrus	−33	18	51	3.1767	31
L superior frontal gyrus	−9	45	48	3.7378	15

The x, y, z coordinates is the primary peak locations in the MNI space. Cluster size > 10 voxels in ANOVA analysis, GRF corrected, voxel *p* < 0.005, cluster *p* < 0.05; Cluster size > 10 voxels in *post-hoc* test, GRF corrected, voxel *p* < 0.005, cluster *p* < 0.05; A+T+, abnormal Aβ_42_ and p-tau; A+T−, abnormal Aβ_42_ and normal p-tau; A−T−, normal Aβ_42_ and p-tau; B, bilateral; L, left; R, right.

### Correlation analysis

**FIGURE 5:**
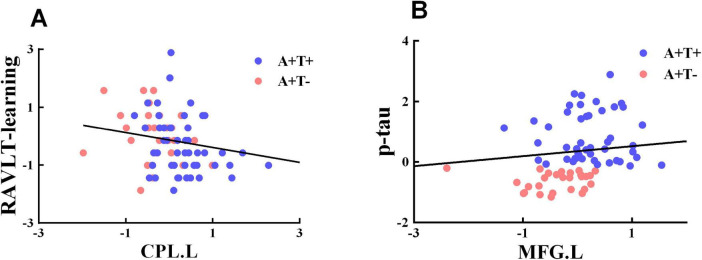
Significant associations between altered functional connectivity of each insula subnetwork and cognitive function. **(A,B**) Age, gender, and years of education were included as covariates of results. CPL.L, left cerebellum posterior lobe; MFG.L, left middle frontal gyrus; A+T+, abnormal Aβ_42_ and p-tau; A+T−, abnormal Aβ_42_ and normal p-tau.

The value of FC of the left CPL was negatively associated with RAVLT-immediate (r = −0.237, *p* = 0.044) and p-tau (r = −0.246, *p* = 0.036) (Figure 5).

### Binary logistic regression analysis

**FIGURE 6:**
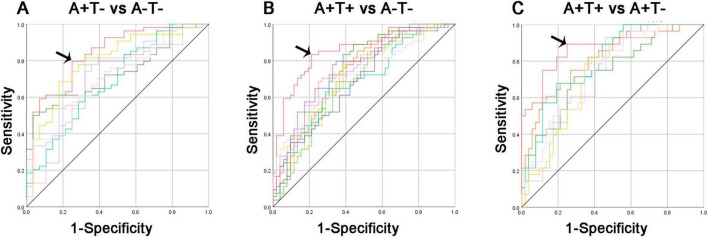
Diagnosis and differentiation of A+T− and A+T+ based on ROC analysis. **(A)** ROC curve showing the classification between A+T− and A−T−; **(B)** ROC curve showing the classification between A+T+ and A−T−; **(C)** ROC curve showing the classification between A+T+ and A+T−. A+T+, abnormal Aβ_42_ and p-tau; A+T−, abnormal Aβ_42_ and normal p-tau; A−T−, normal Aβ_42_ and p-tau. RAVLT, Rey Auditory Verbal Learning Test; p-tau, phosphorylated tau protein.

The ROC curves of each altered index were presented in Figure 6. Obviously, the best-fitting model was the multivariable models (red line), which combined altered FC of each insular subregion. The AUC for distinguishing A+T− from A−T− using the multivariable model was 0.837, with 79.6% sensitivity, and 75.0% specificity (*p* < 0.001). In the group of A+T+ and A−T−, the AUC using the multivariable model was 0.857, with 83.3% sensitivity, and 79.8% specificity (*p* < 0.001). Lastly, the AUC for differentiating A+T+ from A+T− using the multivariable model was 0.882, with 89.3% sensitivity, and 75.0% specificity (*p* < 0.001).

## Discussion

This study aimed to investigate the FC abnormalities of insular subregions in MCI patients with different CSF pathological protein levels and their correlation with cognitive function. The findings revealed that as Aβ_42_ and p-tau abnormalities increased, the FC of insular subregions showed compensatory enhancement, which was associated with cognitive function. Moreover, these abnormalities had significant value in distinguishing between the groups. These discoveries may offer new insights into the pathophysiology of MCI. Clinically, features that provide more accurate and comprehensive information could serve as objective biomarkers, enhancing the ability to diagnose and treat MCI, ultimately improving patient outcomes.

The results of this study indicated that the changes in FC across different insular subregions were similar. As Aβ and tau abnormalities increased, the FC of insular subregions gradually strengthened, which was surprising. According to previous literature, a negative correlation typically existed between tau deposition and FC (Sepulcre et al., 2017; Berron et al., 2020). Previous studies suggested that the loss of connectivity was related to tau-induced progressive structural damage and neuronal death, as tau pathology disrupted axonal stability and impaired axonal transport (Iqbal et al., 2005; Wales and Leung, 2021). Tau-PET studies showed that tau neurofibrillary tangles had adverse effects on brain connectivity in older adults and AD patients, with elevated tau levels linked to weakened intra-network connectivity, a reduction in strongly connected nodes, and decreased global and local network efficiency (Cope et al., 2018; Wisch et al., 2020). However, as research progressed, Quevenco et al. (2020) found that tau pathology was associated with increased activity in more extensive brain regions. They observed that enhanced connectivity between the posterior default mode network (DMN) and areas outside the DMN was positively correlated with overall Aβ levels and local tau accumulation. Moreover, studies proposed that in the preclinical AD process, an initial stage of hyperconnectivity may precede a subsequent stage of diminished connectivity (Schultz et al., 2017). In conclusion, this study revealed a pattern of increased FC in insular subregions as tau pathological burden increased. This phenomenon may involve compensatory neural regulation, early network hyperconnectivity, and potential functional network impairments (Penalba-Sanchez et al., 2022). Future research should further investigate whether these changes represent a transient adaptive adjustment or an early indicator of AD progression, providing new insights for early diagnosis and intervention strategies in the MCI stage.

The results showed a significant increase in the FC between the insula and both the MFG and SFG. The frontal lobe played a critical role in cognitive function, especially in its involvement in the fronto-subcortical circuits (Iaria et al., 2008). Additionally, the MFG and SFG were key regions of the executive control network (ECN) and the DMN, respectively (Liu et al., 2021; Zheng et al., 2024). Notably, the insula is a core region of the salience network (SN) (Xue et al., 2021). The enhanced FC between the insula and these frontal regions also reflected increased FC between the SN and both the ECN and DMN. SN plays a crucial role in responding to biologically and cognitively relevant environmental pressures or events, and it is linked to working memory and higher-order cognitive management (Menon and Uddin, 2010). Within brain networks, the SN is not only an important hub and starting point but also plays a key role in activating brain networks and facilitating the switch between the ECN and DMN during cognitively demanding tasks (Menon and Uddin, 2010; Goulden et al., 2014). In healthy individuals, there was a dynamic balance between the SN and DMN, often showing an inverse relationship (Fox et al., 2005; Sridharan et al., 2008). Although the exact nature of this increased SN connectivity was unclear, one possibility was that diminished inhibitory control may drive the commonly reported lack of task-related DMN suppression associated with Aβ pathology (Sperling et al., 2009; Elman et al., 2016). This disrupted balance may partially explain why hyperconnectivity between the SN and frontal regions was observed in this study. Rather than reflecting a purely compensatory mechanism, this increased FC may indicate reduced network specificity and a breakdown in functional segregation. Thus, the observed hyperconnectivity may represent a compensatory effort by the SN to maintain cognitive functions in the early stages of neurodegeneration, but as pathological burden increases, this mechanism may become inefficient and contribute to cognitive decline. Future studies should investigate whether these FC changes persist or diminish over time and explore their relationship with cognitive performance trajectories.

Additionally, the FC between the insula and the CPL was enhanced and showed a significant association with cognitive function. Specifically, as FC within the cerebellar hemisphere increased, RAVLT-learning scores progressively decreased, indicating that changes in cerebellar FC may be closely linked to declines in learning and memory abilities. This finding was consistent with the study by Aschenbrenner et al., who identified tau pathology as a strong predictor of overall cognitive decline (Aschenbrenner et al., 2018). Other studies similarly showed that patients who were positive for both Aβ and tau experience more severe cognitive decline, aligning with our findings and suggesting that the accumulation of pathological proteins had a significant impact on cognitive function (Kim et al., 2021; Ge et al., 2022). In recent years, researchers have increasingly focused on the role of the cerebellum in cognitive functions. Traditionally viewed as a key regulator of motor coordination, recent studies have demonstrated that the cerebellum also plays a critical role in cognition, emotional regulation, and autonomic nervous system function (Cutando et al., 2022). Numerous prior studies have revealed altered FC in the CPL of MCI/AD patients, underscoring the critical role this region plays in the AD spectrum (Yuan et al., 2023; Xue et al., 2024). These findings highlight the importance of FC between the insula and the CPL, particularly in understanding the pathological mechanisms underlying the AD spectrum. The increased FC between the insula and the CPL may reflect a compensatory response, where heightened connectivity helps maintain cognitive function despite accumulating pathology. This may indicate that the cerebellum and insula work together to compensate for declining cortical efficiency, particularly in memory and executive function tasks. However, as Aβ and tau burden continues to increase, this compensatory mechanism may become insufficient, ultimately leading to network breakdown and severe cognitive impairment. The observed negative correlation between FC of CPL and cognitive performance suggested that, rather than being purely compensatory, hyperconnectivity in these regions may reflect inefficient or maladaptive neural processing. Similar maladaptive hyperconnectivity was reported in other brain networks during the progression from MCI to AD, and excessive connectivity may eventually lead to loss of connectivity as neurodegeneration advances (Xue et al., 2019; Chen et al., 2022; Shu et al., 2022). Thus, investigating the FC between insular subregions and the CPL not only deepens our understanding of the progression of AD but also provides new potential targets for future diagnosis and intervention.

More importantly, the ROC analysis demonstrated the potential of FC-based diagnostic models in early detection and risk stratification of AD pathology in MCI patients. The high AUC values suggested that altered FC in insular subregions could serve as a sensitive neuroimaging biomarker, aiding in the differentiation of MCI subtypes with varying levels of Aβ and tau burden. This is particularly relevant in clinical practice, as early identification of A+T+ individuals is crucial for timely intervention and disease-modifying therapies. Additionally, the multivariable model’s superior performance suggested that combining multiple FC alterations enhances diagnostic accuracy compared to single-region approaches, emphasizing the importance of network-level analyses in AD research. Integrating FC-based diagnostic models with other biomarkers, such as PET imaging and structural MRI, could enhance diagnostic accuracy and reduce reliance on invasive CSF testing. Furthermore, understanding FC alterations may provide insights into disease progression and potential therapeutic targets, paving the way for early interventions that could slow cognitive decline and improve patient outcomes.

## Limitations

Our study had some limitations. Firstly, the study was based on CSF pathological biomarkers, which resulted in a relatively small sample size. However, we applied strict criteria in data processing, which increased the reliability of the results. Future studies should consider validating these findings using a larger sample size. Secondly, CSF pathological proteins in AD lack regional specificity, so we cannot definitively determine whether our findings are driven by localized or whole-brain effects of AD pathology. This limitation may affect the interpretation of how pathological burden influences specific brain regions. In future studies, we plan to incorporate Aβ and tau PET imaging to further investigate the spatial distribution of AD pathology and elucidate its impact on different insular subregions. Thirdly, this study focused only on resting-state fMRI data. In the future, integrating multimodal MRI techniques such as fMRI, DTI, and sMRI may provide deeper insights into the neuroimaging mechanisms of MCI at different pathological protein levels. Additionally, incorporating more dynamic FC analysis methods, such as sliding window correlation analysis, along with task-based fMRI to investigate the dynamic functional changes of insular subregions, may further elucidate their critical role in the progression of MCI.

## Conclusion

This study demonstrated that as Aβ_42_ and p-tau abnormalities increased in MCI patients, cognitive function declines, while compensatory increased in FC of insular subregions occur, indicating enhanced control of the SN over the ECN and DMN. Investigating the changes in FC of insular subregions in MCI patients with different CSF pathological protein levels can provide deeper insights into the neuroimaging mechanisms of MCI.

## Data Availability

The original contributions presented in this study are included in this article/supplementary material, further inquiries can be directed to the corresponding authors.

## Footnotes

1
http://adni.loni.usc.edu

2
www.adni-info.org

3
http://rfmri.org/DPABI

4
http://www.mathworks.com/products/matlab/
